# The anatomy of apathy: A neurocognitive framework for amotivated behaviour

**DOI:** 10.1016/j.neuropsychologia.2017.07.003

**Published:** 2018-09

**Authors:** C. Le Heron, M.A.J. Apps., M. Husain

**Affiliations:** a Department of Experimental Psychology, University of Oxford, Oxford, United Kingdom; b Nuffield Department of Clinical Neurosciences, John Radcliffe Hospital, Oxford OX3 9DU, United Kingdom

**Keywords:** Apathy, Motivation, Decision making, Reward, Anterior cingulate cortex, Ventral striatum

## Abstract

Apathy is a debilitating syndrome associated with many neurological disorders, including several common neurodegenerative diseases such as Parkinson's disease and Alzheimer's disease, and focal lesion syndromes such as stroke. Here, we review neuroimaging studies to identify anatomical correlates of apathy, across brain disorders. Our analysis reveals that apathy is strongly associated with disruption particularly of dorsal anterior cingulate cortex (dACC), ventral striatum (VS) and connected brain regions. Remarkably, these changes are consistent across clinical disorders and imaging modalities.

Review of the neuroimaging findings allows us to develop a neurocognitive framework to consider potential mechanisms underlying apathy. According to this perspective, an interconnected group of brain regions – with dACC and VS at its core – plays a crucial role in normal motivated behaviour. Specifically we argue that motivated behaviour requires a willingness to work, to keep working, and to learn what is worth working for. We propose that deficits in any one or more of these processes can lead to the clinical syndrome of apathy, and outline specific approaches to test this hypothesis. A richer neurobiological understanding of the mechanisms underlying apathy should ultimately facilitate development of effective therapies for this disabling condition.

## Highlights

• A consistent anatomical basis for apathy across neurological disorders.
• Imaging reveals close ties to anterior cingulate and ventral striatum disruption.
• Three core components provide a framework for amotivation linked to these circuits:
• Choosing when to act, sustaining action and learning the value of actions.
• This approach provides testable hypotheses of the mechanisms underlying apathy.

## 1 Introduction

Apathy is a common syndrome that occurs across a range of neurological and psychiatric disorders. Apathy has been conceptualized as a motivational impairment or deficit in goal directed behaviour (Levy and Dubois, 2006) although it is also recognized that loss of motivation may exist in other dissociable domains, e.g. social or emotional apathy (Ang et al., 2017, Lockwood et al., 2017, Robert et al., 2009). Here we focus on apathy as reduced motivation for self-initiated goal-directed behaviour. Current understanding of which components of goal directed behaviour are actually disrupted in apathy is poor and, concomitant with this, there is limited effective treatment for this debilitating syndrome. A richer understanding of the cognitive and neuroanatomical mechanisms that underlie apathy might potentially be important for development of effective therapies in the future. Our approach is first to consider the brain systems implicated in apathy by neuroimaging studies and then relate these to current concepts of the functions of these brain regions.

**Fig. 1:**
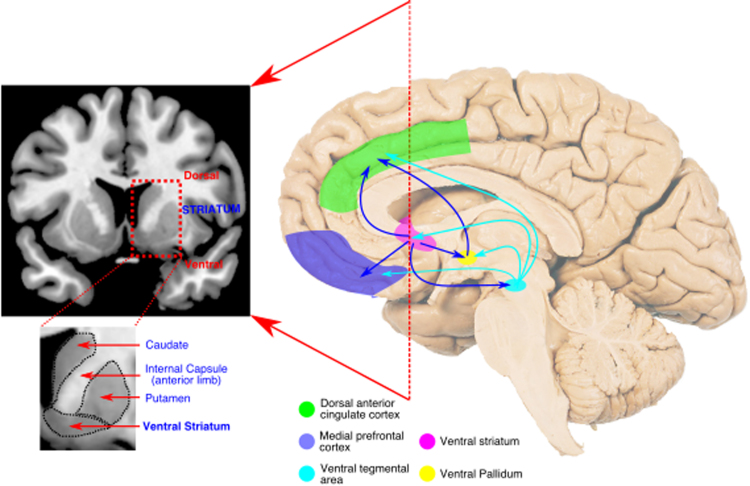
A reciprocally connected network of brain regions for normal motivated behaviour. A network of medial frontal and striatal regions has been strongly implicated in the generation of motivated behaviour in healthy people. Disruption of components of – or connections between – this network is strongly associated with apathy across brain disorders. The dorsal anterior cingulate cortex (dACC) encompasses the anterior cingulate sulcus (Brodmann area 24c & 32). Here medial prefrontal cortex refers to ventromedial prefrontal and orbitofrontal cortex, two anatomically overlapping regions (particularly Brodmann areas 10, 11 and 47) – see Neubert et al. (2015) for a detailed anatomical description.

The last decade has seen the publication of a significant number of studies that report the relationship between apathy and a range of neural markers. Yet, to date, few attempts have been made to synthesize findings regarding the neurobiology *across* different conditions (but see Kos et al., 2016; Moretti and Signori, 2016) and there is particularly limited understanding of the functional anatomy of apathy across disorders. Here, we draw together research across neurological conditions that has used a variety of brain imaging techniques to examine changes in structure and function associated with apathy. This research supports the notion that just as the clinical phenotype of apathy is remarkably similar across diagnostic categories, there are also common brain system alterations across diseases with very disparate underlying pathologies. Our review reveals that, across disorders, there are consistent changes associated with apathy in frontostriatal circuits, and in particular dorsal anterior cingulate cortex (dACC) and ventral striatum (VS), which includes the nucleus accumbens (NAc; see Fig. 1).

Building on these observations we propose a neurocognitive framework for understanding apathy. By leveraging current considerations of the functional roles of dACC and VS from cognitive neuroscience research, we outline the important roles these regions have in motivation and thus the processes that are likely to be disrupted in apathy. Specifically we argue that motivated behaviour requires a willingness to work, to keep working, and to learn what is worth working for. The ACC and VS play vital roles in these three aspects of behaviour and thus provide a viable account of what components of motivation are disrupted in apathy across disorders. This framework provides a testable basis for better understanding the mechanisms underlying apathy, offering clear avenues for future research and targets for therapeutic approaches.

## 2 The anatomy of apathy across disorders

### 2.1 Methods for probing apathy

One of the tools that has been used to explore apathy across a broad range of disorders – including neurodegenerative, traumatic, infectious and genetic conditions – has been neuroimaging. A wide variety of brain imaging techniques has now been employed to probe differences in brain structure and connectivity, perfusion and metabolism, as well as functional properties of specific brain regions. Here, we review studies using such approaches that have reported on anatomical markers of apathetic states. Rather than presenting all studies verbatim, we have endeavoured to select those of the highest quality and which cover the breadth of techniques utilized. We report all results of these studies, before concluding each section with a summary of the main regions implicated.

We have limited this review to neurological disorders in which the prevalence of apathy is relatively high. We do not discuss psychiatric disorders, where there is also a body of work that examines the structural or functional basis of apathy (Foussias et al., 2014, Holroyd and Umemoto, 2016, Treadway and Zald, 2011). As such, this work aims to redress the relative imbalance in the discussion of the neurocognitive mechanisms of loss of motivation in neurological conditions compared to psychiatric ones.

On reviewing this literature we have inevitably had to be selective at times due to the considerable variability introduced by different methods of diagnosis and different levels of controlling for comorbidity (Radakovic et al., 2015, Robert et al., 2009). Questionnaire methods, the most common mode of diagnosis, have been validated to different degrees in each disorder. Furthermore, while some studies have excluded patients with other diagnoses (e.g. depression or cognitive impairment), others have attempted to control for them in analyses. Where such points relating to methodology are pertinent to the discussion we have raised them. Additionally, we have taken a “domain general” approach to the apathy construct. Some apathy measures allow fractionation into components (for example “emotional, cognitive, behavioural”) (Levy and Dubois, 2006) and in some cases authors have reported correlates of these components with imaging analyses. However there currently remains a lack of clarity as to whether these questionnaire-derived components map onto dissociable neurobiological systems. Here, we prefer to initially treat apathy as a generalized construct – a disorder of goal directed behaviour – before later suggesting possible dissociable components based on current neuroscientific understanding of the regions affected. We begin by outlining the evidence for anatomical substrates of apathy in different neurological disorders.

### 2.2 Parkinson's disease

Parkinson's disease (PD) is a common neurodegenerative disorder that leads to widespread metabolic and anatomical changes, and in which a primary dopaminergic deficit is a defining feature (Kalia and Lang, 2015). Apathy is common at all stages of the disease, is associated with impaired quality of life for both patients and their relatives, and is a presenting symptom in a significant number of cases (Aarsland et al., 2009).

#### 2.2.1 Altered metabolism in PD apathy

A number of studies using fluorodeoxyglucose positron emission tomography (FDG-PET) have been conducted in patients undergoing deep brain stimulation (DBS) as a treatment for their PD. The occurrence of apathy after DBS surgery has led to much interest due to the opportunity it provides for potentially understanding the anatomical basis of such effects. In one of the largest and best controlled studies published to date, Robert et al. performed FDG-PET *pre-operatively* in 44 PD patients, and correlated activity with *post-operative* change in apathy scores. Decreased metabolism within the right VS predicted the development of apathy post-operatively, when controlling for the confounding effects related to the DBS stimulation and other medications (Robert et al., 2014, Fig. 2). In non-DBS PD groups, apathy is also associated with altered metabolism within medial frontal brain regions – specifically ACC and orbitofrontal cortex (OFC) bilaterally – as well as temporo-parietal changes (Huang et al., 2013).

**Fig. 2:**
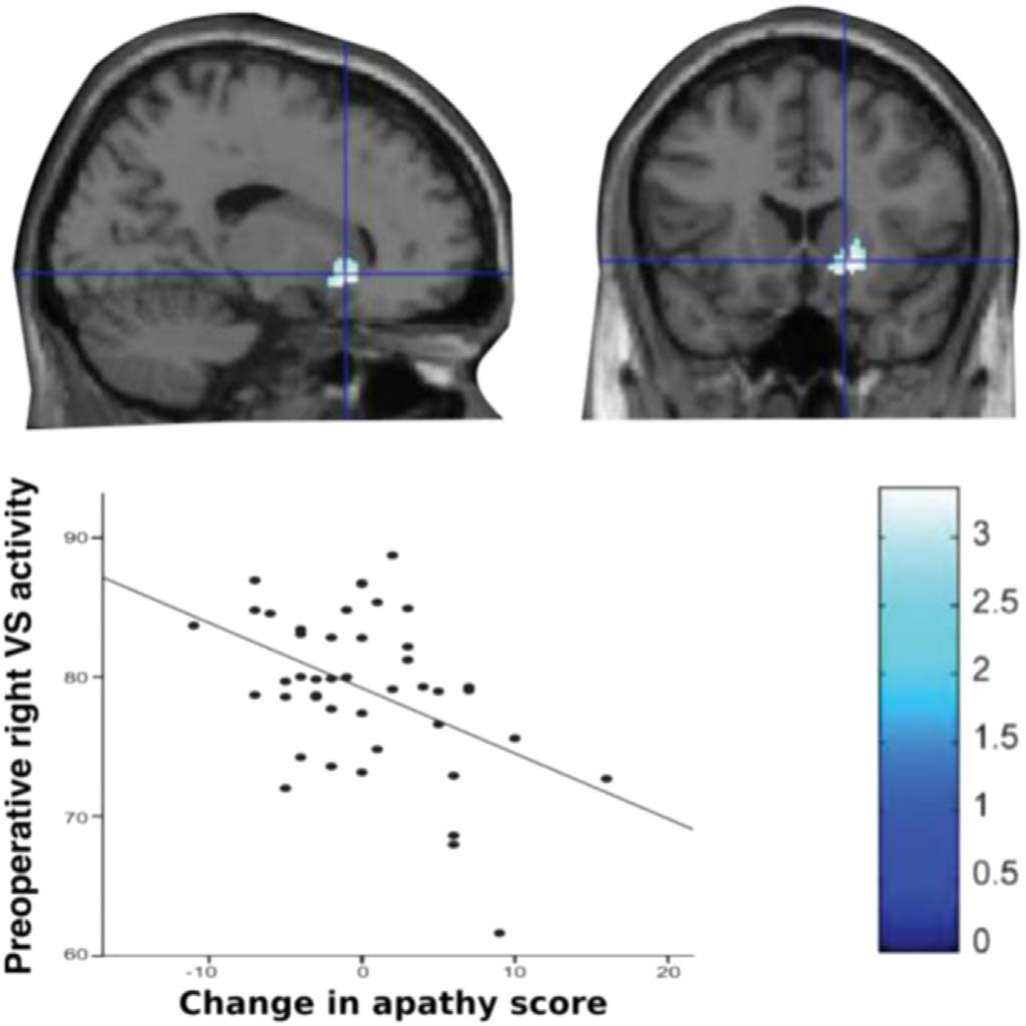
Decreased metabolism within right ventral striatum is associated with greater increase in apathy scores following deep brain stimulation surgery.

*Adapted from*Robert et al. (2014) with permission.

Whilst FDG-PET estimates general metabolic activity (and presumably underlying neuronal loss), research using more specific metabolic imaging techniques has implicated dopaminergic neuromodulatory systems in the development of apathy in PD. Apathetic PD patients have reduced dopamine and/or noradrenaline receptor binding capacity within bilateral VS (Remy et al., 2005). They have reduced striatal dopamine binding capacity even at the time of PD diagnosis, and *before* they have commenced dopaminergic therapy (Santangelo et al., 2015). Furthermore, following administration of methylphenidate they show blunted dopamine release in ACC, OFC, dorsolateral prefrontal cortex (DLPFC), left posterior cingulate cortex (PCC), right temporal cortex and left subcortical regions (particularly thalamus and globus pallidus internal (GPi)), consistent with dopaminergic denervation of these areas (Thobois et al., 2010).

Finally, in a task-based FDG-PET study that hints at the functional significance of these changes, patients with apathy had lower activation of ventromedial prefrontal cortex (vmPFC), striatum, amygdala and midbrain than non apathetic patients when making *actions for incentives compared to no incentives* (Lawrence et al., 2011). The midbrain is the site of the ventral tegmental area (VTA), a key source of dopaminergic projections to the NAc (within VS) and to prefrontal cortex. As we discuss in detail later, a core function of these brain regions might be to represent the *value* of potential actions (see Section 3. Behavioural and Cognitive Neuroscience of Motivation). The blunted incentive-related activity observed in this study suggests altered reward processing in PD patients with apathy may be a consequence of these metabolic changes.

#### 2.2.2 Structural changes associated with apathy in PD

Fewer studies have been performed using magnetic resonance imaging (MRI), but the pattern of results is in line with investigations using FDG-PET. Using a recently developed technique for estimating the three dimensional shape of subcortical structures, investigators found that apathetic individuals had greater atrophy within the NAc and dorsolateral head of caudate, with the strongest result in left NAc (Carriere et al., 2014). In contrast, the authors found no association between *cortical thickness* and apathy status. This is consistent with the largest study to date investigating brain morphology and apathy in PD which found no association between grey matter (GM) volume and apathy status (Baggio et al., 2015). It is worth noting that an earlier study had reported an association between increasing apathy severity and reduced GM volume in a number of brain regions, including anterior and posterior cingulate gyri, insula and lateral inferior frontoparietal regions (Reijnders et al., 2010). However, only a small proportion of patients in this investigation were clinically apathetic.

#### 2.2.3 Functional connectivity is altered in PD apathy

Whilst the metabolic and structural imaging techniques discussed above allow assessment of localised brain regions, complex goal directed behaviour relies on an interactive, distributed network of brain areas. Functional connectivity analysis of resting state functional MRI (fMRI) data provides one estimate of such connections, measuring the correlation of changes in blood oxygen level dependent (BOLD) signal between separate regions. It can be constrained by limiting analyses to brain regions that are anatomically connected. Baggio and colleagues first used probabilistic tractography to parcellate the striata into regions based on *structural* white matter (WM) connectivity with anatomically defined frontal brain areas. They then assessed *resting-state* connectivity between these defined subcortical-cortical areas, and correlated this measure with apathy severity. They reported functional connectivity of frontostriatal circuits was reduced in apathetic cases, specifically affecting connections between medial frontal brain areas (encompassing regions of OFC and ACC) and connected striatal areas (Baggio et al., 2015). These findings are in broad agreement with an earlier, smaller study, which also demonstrated apathy was associated with medial frontal resting state fMRI differences, albeit using a different analysis technique (Skidmore et al., 2013).

#### 2.2.4 Summary

Neuroimaging studies of apathy in PD have implicated VS and dACC, together with regions in medial and lateral prefrontal cortex and the midbrain area that contains the key dopaminergic VTA. All of these regions are interconnected. Furthermore, there are monosynaptic dopaminergic projections from the VTA to each of the frontal regions implicated (Williams and Goldman-Rakic, 1998) – see Section 3.1(a) below. Such widespread involvement of brain regions modulated by dopamine perhaps makes it unsurprising that apathy is so prevalent in PD, a disorder which primarily affects dopaminergic systems. But, as we discuss below, apathy is also very common across a range of brain disorders that traditionally have not been attributed to dopaminergic deficits.

### 2.3 Alzheimer's disease

Alzheimer's disease (AD) is the most common cause of dementia worldwide, and apathy occurs in up to 70% of cases (Mega et al., 1996). As with PD, a range of neuroimaging approaches have been used to investigate the anatomical associations of apathy in AD.

#### 2.3.1 Altered metabolism and perfusion in AD apathy

FDG-PET studies have demonstrated that apathy in AD is associated with reduced metabolism in OFC and ACC, as well as VS, medial thalamus and some temporal regions (Holthoff et al., 2005, Marshall et al., 2007, Schroeter et al., 2011). These results are generally similar to analogous studies in PD, and highly concordant with investigations that have used single-photon emission computed tomography (SPECT) imaging to measure regional cerebral blood flow (which is closely coupled to underlying neuronal metabolism). Research using SPECT has consistently demonstrated that reduced perfusion in ACC and OFC – as well as frontopolar and DLPFC regions in some studies – is associated with apathy in AD (Benoit et al., 2004, Craig et al., 1996, Lanctôt et al., 2007, Migneco et al., 2001, Robert et al., 2006).

Strikingly, when regional metabolism or perfusion is examined *across different disorders* (including frontotemporal dementia (FTD) and subjective cognitive impairment in one study, and AD and organic personality disorder in another), apathy is strongly correlated with reduced ACC activity (Migneco et al., 2001, Schroeter et al., 2011). Furthermore, by controlling for the presence of other behavioural disturbances associated with regional hypometabolism one group identified that hypometabolism in the VTA was specifically associated with apathy, across dementia diagnoses (Schroeter et al., 2011, Fig. 3).

**Fig. 3:**
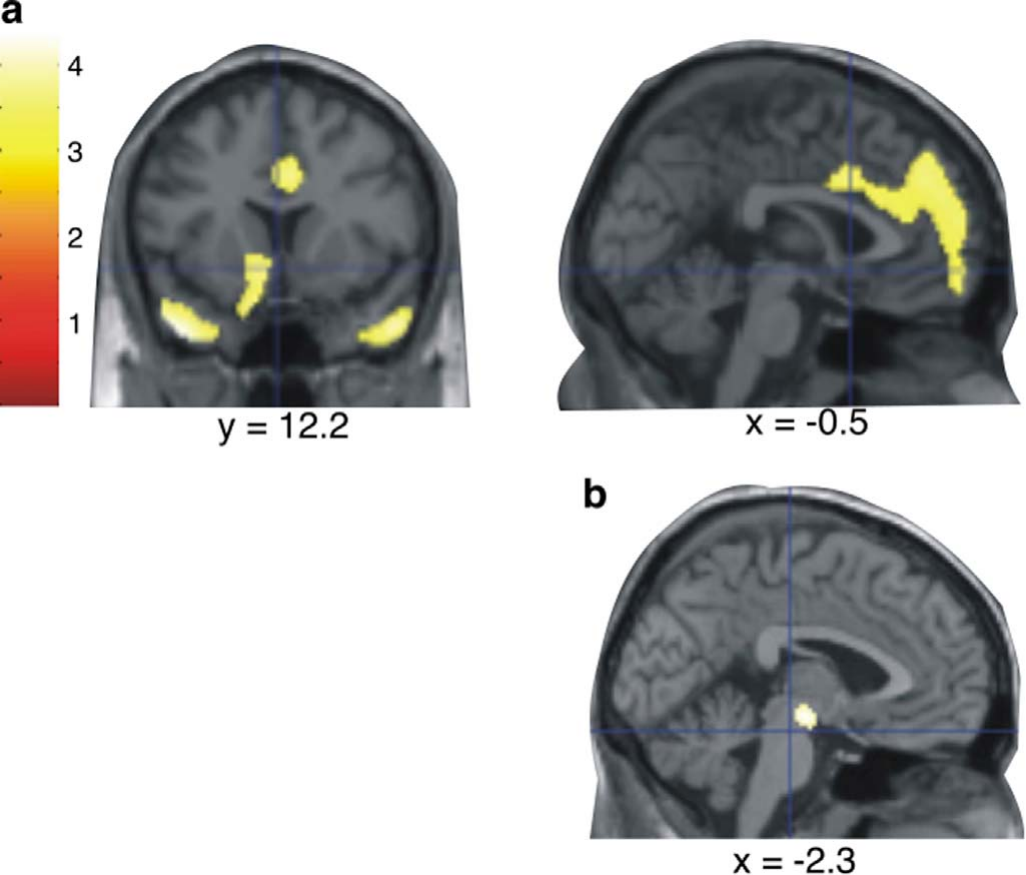
Brain regions in which reduced metabolism was associated with apathy, in a mixed group of patients with dementia (**a**). A disjunction analysis (controlling for other behavioural disturbances associated with altered metabolism) revealed apathy was *specifically* associated with hypometabolism in the region of the VTA (**b**).

*Adapted from*Schroeter et al. (2011) with permission.

#### 2.3.2 Structural changes associated with apathy in AD

As with PD, there is variability in the results of studies examining GM changes and apathy in AD. This is at least in part a reflection of differing inclusion criteria, image acquisition, and analysis techniques. Two well conducted studies – using different analysis methods – identified atrophy within ACC, DLPFC, putamen and caudate nucleus was associated with apathy in patients with AD (Bruen et al., 2008, Tunnard et al., 2011). In contrast, two other investigations of more than 50 patients did not find an association between apathy and GM volume (Kim et al., 2011, Starkstein et al., 2009). It is worth noting that where associations have been identified, volume loss within *medial frontal cortex* is a common feature.

Changes in *WM tracts* connecting brain regions can also disrupt brain function. WM alterations can occur directly as a result of local pathology, or indirectly as a result of pathology within the GM regions the tracts connect. They can be assessed using diffusion-based MRI measures such as *fractional anisotropy* (FA) – the degree that water diffusion within WM fibres is restricted to a particular direction – which reflects integrity of the underlying WM tract (Mori and Zhang, 2006). Using this technique in a population with mild AD, Kim et al. found apathy was associated with reduced FA in the left anterior cingulum (a WM tract that connects limbic structures, including ACC), but no other regions (Kim et al., 2011).

#### 2.3.3 Summary

A wide range of neuroimaging techniques have been used to investigate neural correlates of the apathetic syndrome in AD. Altered functional and structural properties of medial frontal cortex, particularly ACC and OFC, are the most robustly identified cortical changes associated with apathy, whilst subcortically alterations in VS, medial thalamus and VTA are the strongest associations. We note the striking similarity between these regions and those identified using similar techniques in PD.

### 2.4 Other neurodegenerative conditions

#### 2.4.1 Frontotemporal dementia, progressive supranuclear palsy and corticobasal syndrome

Apathy is a core feature of several other neurodegenerative disorders that affect particularly frontal and subcortical brain regions, such as frontotemporal dementia (FTD), progressive supranuclear palsy (PSP) and corticobasal syndrome (CBS) (Litvan et al., 1998, Litvan et al., 1996). It was the most common neurobehavioural disorder in a large imaging study that combined patients with these diagnoses as well as AD (Rosen et al., 2005). The presence of apathy in these groups correlated with reduced GM volume in ACC, OFC, middle frontal gyrus, anterior insula and caudate (Rosen et al., 2005). Two smaller studies limited to patients with FTD also reported atrophy in similar regions associated with apathy, albeit with less robust corrections for multiple comparisons (Eslinger et al., 2012, Zamboni et al., 2008). Furthermore, reduced GM volume in dACC and insula was found in apathetic patients with PSP or AD (N = 17 of each) (Stanton et al., 2013). There was no interaction between diagnosis and apathy in this analysis, suggesting the observed changes were a shared feature of apathy in both conditions. A single study that examined WM tract integrity supports these observations. The authors report that damage to corpus callosum, uncinate fasciculus and superior longitudinal fasciculus is associated with apathy in PSP (Agosta et al., 2014). These WM tracts underlie the GM regions implicated above.

#### 2.4.2 Huntington's disease

Apathy in Huntington's disease (HD) closely tracks disease progression, and is viewed as an ‘intrinsic’ feature of the disease (Thompson et al., 2012). However the few imaging studies to investigate its correlates have been notable for the absence of positive results. A study in pre-symptomatic and early HD did not find any associations between apathy and either GM or WM changes (Scahill et al., 2011). This was a large study (*N* = 240). However, because only individuals with early disease were studied, the rates and severity of apathy were very low. The imaging markers used are also likely less sensitive to early changes than functional approaches. Similarly a diffusion imaging study of 80 patients from another (early stage) demographic, albeit with a greater range of apathy scores, failed to find an association between apathy and FA (Gregory et al., 2015). In contrast, a single, smaller investigation reported FA in the bilateral rectus gyrus WM of HD patients negatively correlated with apathy score (Delmaire et al., 2013). This area contains fibres connecting OFC and subcortical structures, including VS.

#### 2.4.3 Summary

Compared to AD and PD, the number of imaging studies that have investigated correlates of apathy in other neurodegenerative disorders is low, and limited to structural techniques. However, the results from these investigations are generally concordant with those reported for AD and PD, demonstrating that disruption of predominantly medial prefrontal cortex and VS is associated with apathy.

### 2.5 Stroke

Apathy is a consistent and frequent complication of both hemorrhagic and ischemic stroke, affecting approximately one third of cases (Caeiro et al., 2013). Stroke would seem to provide the closest comparison to lesion models in animal work utilized to understand motivated behaviour (reviewed below), albeit with the caveat that background variability in subclinical apathy levels limits the possibility to draw strong conclusions about causality. Nevertheless, apathy following stroke provides an important opportunity to understand the neurobiology of this syndrome. A meta-analysis examining the relationship between stroke and apathy found no lateralizing effects of hemisphere involvement on apathy occurrence, but this study did not attempt to delineate specific brain regions in which apathy was more likely to occur (Caeiro et al., 2013). In fact, the authors actively excluded a number of investigations (summarized below) that have specifically examined this association, to avoid biasing their sample.

#### 2.5.1 Stroke locations associated with apathy

*Subcortically,* basal ganglia lesions, particularly those involving the caudate nucleus or nucleus accumbens, are associated with the development of apathy (Bhatia and Marsden, 1994, Gerace et al., 2013, Maeda et al., 2012, Phillips et al., 1987). Profound apathy is also seen following lesions to the GPi, a crucial output structure of the basal ganglia (Adam et al., 2013). A number of studies report apathy occurring following infarction of the thalamus – specifically paramedian or anterior regions (Engelborghs et al., 2000, Ghika-Schmid and Bogousslavsky, 2000, Krolak-Salmon et al., 2000, Nishio et al., 2011, Perren et al., 2005; reviewed by Carrera and Bogousslavsky, 2006). Limited reports link cerebellar and brainstem involvement with post stroke apathy (Hoffmann and Cases, 2008).

Medial frontal cortex, within the vascular supply of the anterior cerebral artery (ACA), is the classical *cortical* location linked to the development of apathy, or the related disorder of abulia (defined as impaired volition or will). In 100 consecutive ACA territory strokes apathy developed in 43% percent of patients, and was significantly more likely to occur if the lesion included medial regions (frontal pole, cingulate gyrus) or superior frontal gyrus (Kang and Kim, 2008).

#### 2.5.2 Alterations in fronto-striatal circuits may mediate post-stroke apathy

Apathy is also a complication of strokes in other cortical locations (Caeiro et al., 2013). This might be a direct consequence of damage to the brain region, or due to diaschisis of connected regions. Lesions outside the frontal lobes have been associated with altered frontal metabolism (measured using MR spectroscopy of the frontal poles) in apathetic compared to non-apathetic patients (Glodzik-Sobanska et al., 2005). Similar evidence for the disruption of frontal functional networks is provided by an in-depth case study of a single patient who developed apathy following multiple small embolic infarcts. Despite structural imaging demonstrating the ACC was not primarily affected by the emboli, the patient had reduced functional connectivity (assessed using resting state fMRI) within the cingulo-opercular network, providing further evidence of the occurrence of apathy associated with frontal network dysfunction *at a distance* (Siegel et al., 2014). One study has in fact concluded that cerebral blood flow within the basal ganglia is reduced in apathetic compared to non-apathetic patients, irrespective of stroke location (Onoda et al., 2011). Finally, a recent report of patients who had suffered ischemic stroke used diffusion-based MR imaging and graph theory analysis to estimate structural WM connectivity between different brain areas and derive a network associated with apathy. The authors demonstrated that reduced connectivity in many regions remote from the stroke, including the same frontal and basal ganglia regions outlined above, was associated with apathy (Yang et al., 2015).

#### 2.5.3 Summary

Apathy is a recognized feature of strokes that affect portions of the medial frontal cortex including the ACC, as well as NAc, ventral pallidum, caudate nucleus, and medial thalamic nuclei. Although it also commonly occurs in association with more distant lesions, some evidence suggests that this may be mediated by alterations within fronto-striatal structures.

### 2.6 Cerebral small vessel disease

Apathy frequently accompanies both sporadic and hereditary cerebral small vessel disease (CSVD) which tends to affect subcortical and deep WM regions of the brain (Ligthart et al., 2012, Reyes et al., 2009). From a neurobiological perspective it is important to understand whether this is driven by damage to particular WM tracts, or whether it is simply a more general effect. A recent large study of sporadic CSVD addressed this question using diffusion MRI. Apathy was associated with reduced FA within WM pathways including the anterior cingulum, uncinate fasciculus and fornix (Hollocks et al., 2015). These white matter tracts link brain regions such as ACC and VS which are strongly implicated in the development of apathy in other neurodegenerative conditions, reviewed above.

Intriguingly, CSVD may also be an important modulator of apathy in other neurodegenerative conditions. This is illustrated by two large studies of people with different dementia diagnoses. They reported apathy was associated with the presence of both white matter hyperintensities (WMH - the pathognomonic imaging marker of CSVD) (Jonsson et al., 2009) and lacunar infarcts within deep WM (Lavretsky et al., 2008). Additionally, within AD patients, even after excluding lacunar infarcts (and therefore many subjects who likely had CSVD), Starkstein et al. still found the presence of apathy was associated with greater *frontal* WMH volume (Starkstein et al., 2009).

### 2.7 Human immunodeficiency virus infection

Apathy has been recognized as a significant accompaniment of human immunodeficiency virus (HIV) infection, and is thought to arise from the direct result of viral infection, which has a particular affinity for the basal ganglia, rather than as a reactive process (McIntosh et al., 2015). Apathy in HIV is associated with reduced NAc volume (Paul et al., 2005). As is evident in other disorders, there is disruption of frontal WM tracts in apathetic individuals with HIV. Two studies have demonstrated apathy is associated with reduced FA within the genu of corpus callosum, anterior thalamic radiation and superior/anterior corona radiata – tracts that connect medial frontal cortex and subcortical regions (Hoare et al., 2010, Kamat et al., 2014). Although limited in scope, the imaging correlates of apathy in HIV infection are consistent with changes observed in other conditions, particularly those observed in VS.

### 2.8 Traumatic brain injury

Aside from isolated case reports, few studies have examined the neural correlates of apathy, a frequent complication of head injury (Arnould et al., 2013). In a heterogeneous group of 60 patients who had suffered traumatic brain injury (TBI), reduced volume within brain regions including medial frontal cortex was associated with greater severity of apathetic features (Guild and Levine, 2015). Another small study of 10 apathetic TBI patients reported that volume loss in the left hippocampus strongly correlated with apathy (Takayanagi et al., 2013). Overall*,* evidence for the neuroimaging correlates of apathy in TBI is limited, and further research is required to determine whether the associations described for other conditions are also true for this complex entity.

### 2.9 Summary: common themes across diseases

**Fig. 4:**
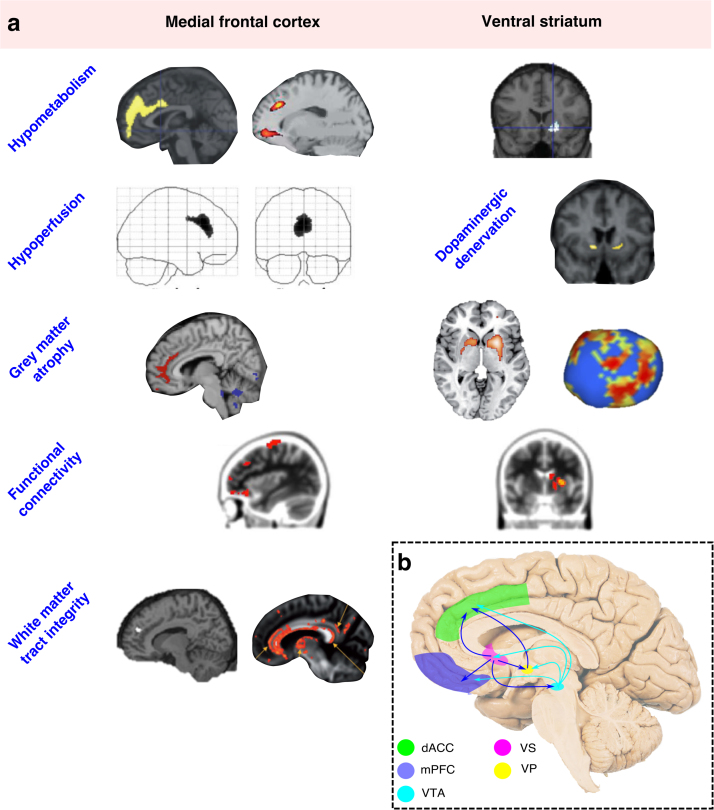
**Consistent anatomical associations with apathy across imaging modalities and underlying disorders.** Changes affecting dorsal anterior cingulate cortex and ventral striatum are particularly prominent **(a)**. A connected system of brain areas consistently associated with apathy, which form an interconnected network within the medial forebrain **(b)**. *dACC – dorsal anterior cingulate cortex; mPFC – medial prefrontal cortex; VTA – ventral tegmental area; VS – ventral striatum; VP – ventral pallidum. Images (from top left) adapted with permissions from:* (Huang et al., 2013, Robert et al., 2014, Schroeter et al., 2011; Migneco et al., 2001, Remy et al., 2005, Stanton et al., 2013; Baggio et al., 2015, Bruen et al., 2008, Carriere et al., 2014; Hollocks et al., 2015, Marshall et al., 2007, Ota et al., 2012).

Across a range of underlying pathologies, and despite variations in design, neuroimaging studies that have investigated the anatomical correlates of apathy converge on a consistent pattern **(**Fig. 4**)**. Broadly, apathy is associated with disruption of medial frontal cortex – in particular the ACC and OFC – and subcortical structures including the VS, medial thalamus and VTA, or connections between these regions. These associations are demonstrated across techniques that measure underlying neuronal metabolism, GM atrophy, and both structural and functional connectivity.

We note that whilst dACC and VS are identified most consistently across disorders, other regions are also implicated less regularly. Interestingly these regions, including insula, DLPFC and OFC are mono-synaptically connected with each other, and also to the dACC and VS. This suggests that disruption to the wider circuitry connected to dACC and VS may also be linked to apathy. One possible explanation for the less consistent involvement of these areas – compared to dACC and VS – may be that they are linked to different dimensions of apathy. However, this possibility has not yet been rigorously examined, in part because many (although not all) apathy measures do not allow for the dimensional structure of apathy to be explored.

Overall, disruption of these structures – particularly dACC and VS – is a unifying feature across the majority of studies. This suggests a strong anatomical basis for the disrupted goal directed behaviour that defines the apathetic syndrome, and allows us to consider the functional significance of these brain regions for apathy.

## 3 Behavioural and cognitive neuroscience of motivation: trading off benefits with costs

Taken together, it is clear from neuroimaging studies across several different disorders that apathy is most commonly associated with changes in the metabolic, gross and functional anatomy of fronto-striatal circuits. Moreover, the most common deficit appears to be to VS, and to portions of interconnected regions of the ACC, lying in the sulcal banks of the mid and anterior cingulate cortex.

The dorsal ACC is comprised of multiple different sub-regions that can be delineated anatomically (Devinsky et al., 1995, Neubert et al., 2015a) and functionally (Apps et al., 2016). Strikingly there are distinctions in anatomical and functional properties along two axes. Firstly, a rostral-caudal axis, with more anterior portions containing less dense projections to the motor system and playing more important roles in cognitive processes, such as decision-making (Silvetti et al., 2014). Secondly, there are also striking differences between the sulcal and gyral portions of the ACC (Apps et al., 2016). In this axis, the defining distinction in function is that the gyral portion of the ACC appears to have a quite specific role in processing social information, whereas the sulcal region seems to be more specialized for processing motivationally relevant information that guides one's own behaviour. This would suggest that apathy may be linked to the functional properties of the sulcal ACC, rather than the gyral portion.

Other regions that are implicated also strongly connect to similar portions of the ACC and VS. These include the amygdala, anterior insula, DLPFC, VTA, and more ventral portions of the medial prefrontal cortex (Balsters et al., 2016, Beckmann et al., 2009, Haber et al., 1995, Mesulam and Mufson, 1982, Mufson and Mesulam, 1982; Neubert et al., 2015; Petrides and Pandya, 1999; Tighe et al., 2012; Vogt, 2009; Yeterian and Pandya, 1991). However, the dACC and VS appear to be the core regions to which disruption in normal functioning leads to apathy (Kos et al., 2016, Theleritis et al., 2014, Wen et al., 2016).

Why might damage to these brain regions lead to apathy? In the next section, we discuss the anatomical and functional properties of the dACC-VS circuit based on the most current considerations from cognitive neuroscience studies. The evidence points to the important role these regions have in three key components of motivation: willingness *to initiate* an action (to work), *to sustain* performance (to keep working), and *to learn* whether actions are worth performing (what is worth working for) (Holroyd and Yeung, 2012; Kolling et al., 2016b; Salamone et al., 2016). Within the context of a cost-benefit decision-making framework, these regions are considered to play a key role in “valuing” the performance of actions and thereby motivating behaviour. Before discussing these accounts, we first outline the anatomical properties of these regions across species.

### 3.1 (a) ACC – ventral striatal – pallidal loop and modulation by VTA

Classical accounts suggested that basal ganglia nuclei formed closed loops with cortical areas via the pallidum, leading to the notion of distinct ‘fronto-striatal’ circuits (Alexander and Crutcher, 1990, Haber et al., 1993). Such proposals have since been shown to be too simplistic because it is now evident that these loops are not isolated, but also connect to some extent with each other, through both cortical and sub-cortical regions. Nevertheless, there is strong evidence that neurons in the VS have monosynaptic projections to the ventral pallidum (VP) (Haber et al., 1995, Haber et al., 1993). This part of the VP projects to the dorsal and ventral bank of the dACC sulcus, and in addition these same regions project to the core and shell of the NAc as well as neighbouring portions of the VS (Vogt, 2009, Yeterian and Pandya, 1991). Thus, parts of the VS forms loops with the dACC, via the VP. The components of this loop are likely to have similar functional properties and damage in any one of these regions may have consequences on the same behavioural domain. Notably, both the ACC and VS also receive strong, monosynaptic inputs from dopaminergic neurons in the VTA. Dopamine has often been linked with motivated behaviour and the processing of rewards (Berridge and Kringelbach, 2015, Wise, 2006). Increasing levels of dopamine increases incentivisation by rewards, and also increases the willingness to overcome effort costs (Chong et al., 2015, Manohar et al., 2015, Salamone et al., 2016). These anatomical considerations point to the possibility that motivating behaviour relies also on dopaminergic projections from the VTA to ACC and VS.

### 3.1 (b) Behavioural neuroscience and a circuit for motivation

A complexity inherent in understanding the mechanisms of apathy based on neurological populations is that anatomical damage is not circumscribed to gross or cytoarchitectonic anatomical boundaries. However, focal experimental lesions to regions of ACC (areas cg1 and 2 - considered homologous to dACC in humans) and VS in rodents (Vogt and Paxinos, 2014), lead to changes in motivation and may also induce “apathy” in rodents.

**Fig. 5:**
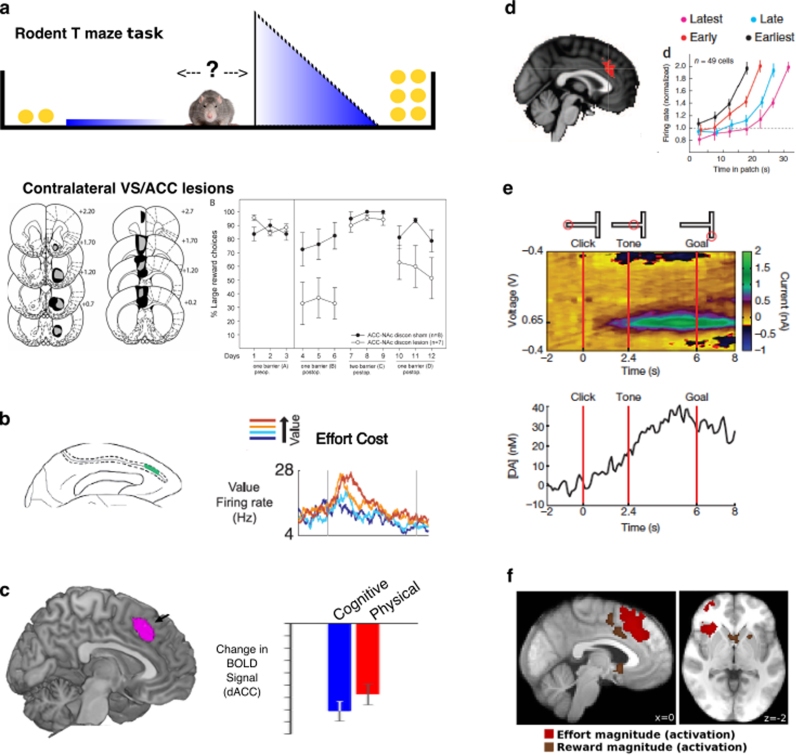
**Importance of ACC and VS for motivated behaviour.** **Effort based decision making.** Lesions to ACC, VS or crossed ACC/VS disconnection lesions (pictured) cause rodents to choose more low effort/low reward options, without altering their preference for high rewards options if effort costs are equal **(a)** (from Hauber and Sommer, 2009). Single neurons within macaque ACC modulate firing rates to the net value of a choice (reward – effort) **(b)** (from Kennerley et al., 2009). BOLD activity within human ACC signals the subjective value of a choice (reward – effort), whether the cost is physical or cognitive **(c)** (from Chong et al., 2017) **Modulating behavior to foreground and background environment characteristics.** BOLD activity correlates with the value of foraging (switching from current option), rather than the value of the actual chosen option **(d – left panel)** (from Kolling et al., 2012). Single neuronal recordings from the ACC of macaques performing a patch leaving foraging task show earlier increases in firing rate on trials where the animal leaves the patch faster **(d – right panel)** (from Hayden et al., 2011b) **Sustaining behavior towards a goal.** Recordings from rodent VS show ramping dopamine signals as the animal approaches a rewarding outcome, thought to drive persistence of behaviour towards the goal **(e)** (from Howe et al., 2013) **Monitoring outcomes of actions.** ACC and VS activity encodes information on both reward and effort magnitudes during the outcome phase of an action **(f)** (from Scholl et al., 2015) (all figures reproduced with permissions).

A striking and oft noted effect of lesions in either region is an alteration in the willingness to engage in a task (Hauber and Sommer, 2009, Salamone et al., 2016, Walton et al., 2006, Walton et al., 2002). Over the last decade emerging evidence supports the hypothesis that it is not simply reduced incentivisation by rewards, but a reduction in willingness to work to obtain them. In particular, rodents still show a preference for higher (compared to lower) reward magnitudes after lesions to ACC or VS, but not if those rewards are associated with a cost of exerting effort (Hauber and Sommer, 2009, Salamone et al., 2016, Walton et al., 2006) (Fig. 5a). Lesions to either ACC or the VS therefore appear to reduce the willingness to exert effort to obtain rewards and thus, create apathetic states in rodents. There is not scope to fully review this literature here, but there is now an overwhelming wealth of behavioural evidence which highlights the crucial role of the ACC-VS loop in motivating the exertion of effort towards a goal, sustaining this effort and in learning whether a behaviour is worthwhile (Salamone et al., 2016).

In the following sections we outline factors that have emerged from cognitive neuroscience research which appear to be crucial for motivated behaviour. Although not explicitly noted in the field, these factors relate to three different phases of behaviour:

• Choosing whether to perform an action or a series of actions
• Performing and sustaining motivation for a behaviour, and
• Learning, through outcome monitoring, whether a behaviour was worth performing.

For each, we discuss neuroimaging research and where appropriate, non human primate neurophysiology, which highlights the roles of the ACC and VS in these processes.

### 3.2 ‘Is it worth it?” Cost-benefit evaluation for exerting effort

A key component of motivation appears to be that prior to performing a behaviour people engage in a cost-benefit evaluation (Phillips et al., 2007, Salamone et al., 2016). Is the behaviour worth performing or is an alternative option better? As highlighted in the section above, one of the important costs that influences behaviour is the amount of effort that needs to be exerted to obtain a goal. Cognitive neuroscience research has placed emphasis on the idea that decisions of whether to exert effort are made by ascribing a numerical ‘value’ to exerting effort to obtain rewards. The cost of exerting effort devalues the potential reward on offer (Chong et al., 2017, Hartmann et al., 2013, Klein-Flügge et al., 2016). Neuroimaging studies in healthy human adults have consistently implicated the dACC and VS in signaling the effort costs of a particular behaviour, but also in signaling rewards on offer prior to any action being performed (Apps and Ramnani, 2014, Botvinick et al., 2009, Croxson et al., 2009, Schmidt et al., 2012, Vassena et al., 2014). Neurophysiological recordings from macaque monkeys have also identified neurons in the ACC, the firing of which relate to cues that predict rewards on offer as well as effort costs required (Kennerley et al., 2009) **(**Fig. 5**b).** The ACC and VS are therefore sensitive to effort and reward related information *preceding* performance of a behaviour. Thus these regions are well placed to evaluate the cost and benefits of exerting effort, and indeed activity of some ACC neurons correlates with an integration of these two key factors (Kennerley et al., 2009).

More recent work has also begun to examine the neural mechanisms that guide *decisions* of whether it is worth exerting effort. Some fMRI studies have required people to make choices between different ‘offers’ which vary in terms of the reward on offer and also the amount of effort required. Using computational modeling of the choices each person makes, researchers can quantify the extent to which each individual is devaluing rewards by the required effort. Moreover, such models provide an estimate of the subjective value (*SV*) of exerting effort for reward. That is, they can examine how much value is ascribed to each effort-reward combination and thus how motivated an individual is to choose to put in effort for a reward. Using this approach several investigations have now shown that the ACC and VS signal *SV* of exerting effort, a computation that is considered to be important in guiding motivation and in making decisions of whether to exert effort (Burke et al., 2013, Klein-Flügge et al., 2016, Kurniawan et al., 2010, Massar et al., 2015, Scholl et al., 2015, Skvortsova et al., 2014).

The ACC - and also the VS - seems to be sensitive to the *SV* of exerting effort regardless of the type of task and thus the form that the effort takes. In the real world, we exert effort in tasks that can be physically or mentally demanding (Apps et al., 2015, Kurzban et al., 2013, Verguts et al., 2015, Westbrook and Braver, 2016). Thus, we must decide whether it is worth exerting effort in different domains of behaviour, be they physically or cognitively demanding. Recent studies have shown that regardless of whether effort is cognitive (in the form of difficulty of perception) or physical (grip force), activity in the ACC and VS signals the expected effort and reward level (Schmidt et al., 2012). Chong and colleagues asked people to make effort-based decisions when the costs were either switches of peripheral attention or physical (grip force). They found that the ACC, as well as the anterior insula and DLPFC, signaled the *SV* of devalued rewards *for both types of cost* (Chong et al., 2017) **(**Fig. 5**c)**. Taken together, these results suggest that the ACC and VS may play an important domain-general role in subjectively evaluating whether it is worth exerting effort into a task.

Although SV signals are present in the ACC and VS regardless of the form of the effort, activity does appear to be specific to effort costs rather than also other forms of cost that devalue rewards. Studies that have directly compared processing of the *SV* of rewards associated with effort, temporal delays or risk, consistently report that activity in the ACC signals the *SV* of effort costs. When associated with temporal delays or risk, *SV* is processed in other frontal lobe regions but not in the dACC (Burke et al., 2013, Massar et al., 2015, Prevost et al., 2010).

**Fig. 6:**
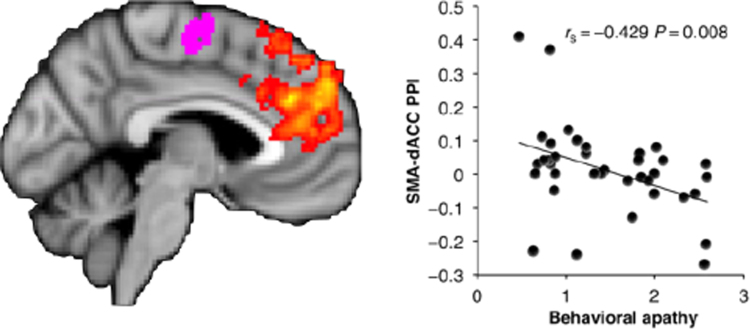
Level of functional connectivity between dorsal ACC (yellow-orange) and supplementary motor area (purple) predicts behavioural apathy scores in healthy subjects (higher score corresponds to greater apathy traits) *(from*Bonnelle et al., 2016*with permission).*

Given the important role of ACC and VS in domain-general decisions about whether to exert effort, an obvious question is whether activity in these regions differs depending on levels of apathy. In a recent study Bonnelle and colleagues (Bonnelle et al., 2016) examined precisely this question by asking healthy people to perform a physical effort-based decision-making task. In line with other studies, they reported that, among other regions, activity in the ACC was related to the willingness to exert effort. In addition, activity in this region, and its structural and functional connectivity with medial regions involved in motor control (such as the supplementary motor area), was correlated with self-report measures of apathy **(**Fig. 6**)**. Thus, in healthy people, activity in the ACC may be related to computing the value of exerting effort, with apathetic people overly sensitive to effort costs and devaluing rewards to a greater degree, associated with levels of activity in the ACC.

Consistent with the behavioural outcomes of the lesion work described in the previous section, human ACC and VS may play crucial roles in valuing and motivating choices that will result in the exertion of effort. Increased sensitivity to effort associated with alterations in response within the ACC and VS may lead to reduced levels of motivation and thus apathy. Moreover, effort-based decision-making may provide a fruitful, neurobiological framework for understanding the mechanisms of apathy.

### 3.3 Sustaining motivation, opportunity costs and foraging for alternatives

To obtain many rewards or goals in the world, it is not sufficient to simply decide to embark on a course of action. We must also sustain motivation throughout extended sequences of behaviours (Holroyd and McClure, 2015, Holroyd and Yeung, 2012, Shenhav et al., 2013, Verguts et al., 2015, Westbrook and Braver, 2016). Recent accounts have argued directly that the ACC is involved in guiding decisions relating to the value of switching *away – or sticking to –* a current planned sequence of action (or cognitive process) (Kolling et al., 2016a, Kurzban et al., 2013). The ACC and connected regions such as the VS may therefore guide choices to embark on an action towards a goal (as outlined above), and then sustain the motivation required to persist with the behaviour until the goal is reached.

There are two key aspects of such accounts. First, activity should be present in regions involved in sustaining motivation during the performance of a sequence of behaviours towards a goal or outcome, not just at the time of decisions to embark on behaviours or at the receipt of outcomes. Second, activity should be sensitive not only to the value of an ongoing action during its performance, but also to the value of alternative courses of action or other features of the environment that would effect the willingness to continue with a current behaviour. This is because motivation to sustain effort in the current task should depend also upon alternative opportunities being less favourable. Such ‘opportunity cost’ theories propose that if the value of what is being performed is less than the background average of alternatives, people should stop doing what they are doing and switch (Kurzban et al., 2013; Niv et al., 2007).

Crucially there is evidence that activity in both the ACC and VS is sensitive to both of these two properties. Recordings in monkeys have identified neurons in the ACC that code for the value of “rejected” offers after choices have been made, and also neurons that code the value of chosen options relative to unchosen options (Blanchard et al., 2015, Blanchard and Hayden, 2014). Neurophysiological and neuroimaging studies have also shown that activity in the ACC tracks information about the value of a *current* plan, relative to alternatives, during extended sequences of actions that require persistence and effort in order to obtain desired outcomes (Blanchard et al., 2015, Croxson et al., 2009, Kolling et al., 2016b, McGuire and Kable, 2015, Wittmann et al., 2016) **(**Fig. 5**d)**.

Signals in the VS are also present during extended sequences of behaviour. Of particular interest given the close association between apathy and disrupted dopaminergic systems, tonic dopamine levels in the VS ramp up when an animal travels towards rewards (Hamid et al., 2016, Howe et al., 2013) **(**Fig. 5**e)**. Moreover, tonic dopamine levels in the VS also track information about the average reward rate in an environment – a key determinant of the relative value of a current course of action, and the vigor with which it should be pursued (Niv et al., 2007).

A closely linked line of research has used the frameworks of foraging, from the field of ecology, to precisely quantify the factors that influence switching behaviours. *Foraging theories* describe how key features about alternative options (or ‘background environment’) and one's current behaviours (the foreground or ‘patch’) influence the relative value of a current course of action (Charnov, 1976, Kolling et al., 2016b). Recently, a number of studies have, in one form or another, provided support for the proposal that activity in the ACC is linked to key aspects of foraging behaviours. Neurons in the ACC signal the “reward rate” within a patch, a key factor that determines how motivated an individual should be to stick with a current behaviour or switch to another (Hayden et al., 2011b). The results of several neuroimaging studies support this finding, suggesting that a key feature of the ACC signals may be integration of information about the (background) environment and an individual's current behaviours (Kolling et al., 2016a, Wittmann et al., 2016).

Taken together, there is growing evidence that signals in both ACC and VS are modulated by the value of a chosen action *during* sequences of actions, as well as crucially the value of alternatives and other properties of the environment. Such factors influence the willingness to continue with a course of action, suggesting that the functional properties of these regions might have a direct link to motivational impairments when disrupted in neurological disorders.

### 3.4 Outcome monitoring: learning the value of being motivated

When a behaviour has been executed, outcomes reveal the success or failure of the course of action and thus its actual value. A key theme of several theories of ACC and VS function is that these regions play a prominent role in monitoring the outcomes of our actions (Carter et al., 1998, Kolling et al., 2016b, Schultz, 2013, Schultz and Dickinson, 2000a). Most theoretical accounts suggest that both regions process outcome-related information under the principles of *reinforcement learning theory*. That is, activity in both regions encodes the expected value of a course of behaviour, and when outcomes are revealed, the actual value of the outcome is compared to this expectation. When actual and expected outcomes are discrepant in value, prediction errors (PEs) code the size of the discrepancy and allow future estimates of the value of acting to be updated.

Crucially such PEs allow for one to optimally motivate behaviour by learning which behaviours will likely result in beneficial outcomes. There is now a wealth of neurophysiological and neuroimaging evidence that activity in the VS and ACC signals PEs (Hayden et al., 2011a, Holroyd and Coles, 2008, Jahn et al., 2016, Jocham et al., 2009a, Lockwood et al., 2016, Matsumoto et al., 2007a, McClure et al., 2003, O’Doherty et al., 2003, Rushworth and Behrens, 2008; Schultz and Dickinson, 2000). These regions may therefore play vital roles in guiding people's learning from the outcomes of their behaviour, and thus learning which behaviours to be motivated to perform in the future.

More recent accounts have suggested that whilst PE signals in the VS guide learning of explicit stimulus-outcome associations, PEs in ACC may in fact be more related to *updating the value of actions* or, even more broadly, general levels of motivation. PEs in ACC are often present when the value of performing actions are updated, but are less commonly found when stimulus-outcome association values are updated (Jocham et al., 2009a, Matsumoto et al., 2007a). Furthermore, impairment in learning action – but not stimulus – values has been demonstrated in humans with dorsal ACC damage (Camille et al., 2011). Some researchers have also reported effort-related PEs in the ACC. When learning which actions are associated with higher or lower levels of effort, PEs in ACC drive the learning, allowing individuals to minimize costs incurred when obtaining beneficial outcomes (Scholl et al., 2015, Skvortsova et al., 2014). Very recent evidence also suggests that outcome related signals putatively originating from ACC are impaired after dopaminergic lesions even when performance on a task requiring the monitoring of outcomes is unimpaired (Wilson et al., 2016).

Thus, abundant evidence points to the VS and particularly the ACC in coding information about the outcomes of our actions, which can in turn drive learning about what behaviours are worth performing.

## 4 A framework for apathy: subjective cost-benefit valuation, sustained motivation and outcome monitoring

We have presented evidence that apathy is associated with disruption to a VS-ACC circuit and connected brain regions, including OFC, VP and VTA. We have also outlined the current understanding of the role of these areas in normal motivated behaviour. Using this as our basis, we propose that apathy can be best thought of as a deficit in cost-benefit evaluation that will be present in three different phases of behaviour, when:

• Choosing whether to pursue a behaviour
• Persisting with a behaviour
• Evaluating and learning the costs and benefits of acting.

In this section we put this forward as a framework for understanding apathy across diverse pathologies and highlight falsifiable predictions based upon it **(**Box 1**)**.

Box 1 **Future Directions.**

Based on the research presented in this review, we predict that three different phases of motivated behaviour may be disrupted in patients with apathy. These are engaging in, persisting with and learning from actions towards goals. To date, most elements of the framework we have outlined have not been rigorously tested, and thus, there are a number of possible avenues of research to pursue. Specifically:

• Is apathy across brain disorders characterized by reduced willingness to exert effort, and is this driven by over-weighting of effort costs, under-weighting of reward, or a combination of these mechanisms?
• Can different aspects of apathy (as currently identified by some questionnaires – e.g. Ang et al., 2017; Sockeel et al., 2006)
  be mapped to different components of our proposed framework?
• Are apathetic patients less likely to persist in tasks than non-apathetic ones?
• Do apathetic individuals weigh information about the background environment or alternative courses of action differently from non-apathetic ones?
• Are effort costs learnt differently by apathetic patients?
• Does stimulation of the ACC-VS loop lead to changes in effort processing, persistence or outcome learning?
• How do connections between the ACC-VS and other regions modulate motivation and relate to apathy?
• Do different aspects of motivated behaviour and apathy (e.g. engagement, persistence, learning) map onto specific neuromodulatory systems?

### 4.1 “Is it worth it?” Willingness to work and exert effort

Apathetic patients may be less likely to engage in effortful behaviours, in much the same way that rodents with ACC lesions, or following dopamine depletion of VS, are biased away from high effort actions (Salamone et al., 2007, Walton et al., 2002). This alteration in behaviour could be related to a reduction in *value* of such options, as a consequence of either a greater cost attributed to required actions, reduced incentivisation by the rewarding outcomes of the action, or an integration of both these factors. Specifically, this framework predicts that some apathetic patients will show altered effort-based decision making, rejecting more beneficial, but potentially more costly actions, in favour of actions that require less effort.

Such behavioural consequences can be well characterized in effort-based decision-making tasks. Emerging evidence already supports the utility of this approach. Altered effort based decision making has been related to apathy in healthy subjects and in people with schizophrenia (Bonnelle et al., 2016; Hartmann et al., 2015) **(**Fig. 6**)**. Intriguingly, there is also evidence that self-initiation and social domains of apathy relate to the willingness to put in effort to benefit ourselves or other people, respectively (Lockwood et al., 2017). Furthermore, dopamine modulates willingness to exert effort for reward in non-apathetic patients with PD (Chong et al., 2015). Blunting of physiological responses to reward has also been demonstrated in PD patients with apathy (Muhammed et al., 2016). With these sorts of tools, future research will be able to probe apathy, across different diagnoses, using effort-based decision-making to test this framework. This approach also has potential to evaluate the effectiveness of any therapeutic interventions in terms of re-weighting people's subjective valuations of cost-benefit choices.

### 4.2 “Should I persist?” Motivated behaviour towards a goal, and tracking of alternative options

Given the important role of ACC and VS for persisting through sequences of behaviours, we predict that some apathetic patients will be differentially sensitive to the value of their current behaviour, *relative* to alternatives or contextual information about the environment. Specifically, these patients will (i) give up quicker during tasks, (ii) weigh background environment relative to their current activities differently from healthy people and (iii) show reduced invigoration during sequences of behaviour. Although to date there is currently limited evidence in favour of these hypotheses, this has generally been due to an absence of studies using neuroscience or computational frameworks to probe apathy.

### 4.3 “Was it worth it?” Effects of learning on behaviour

Finally, we have discussed evidence that ACC, VS and related regions may play a critical role in updating of values associated with actions and rewards by signaling prediction error. Our model suggests that apathetic behaviour in some individuals might arise if learning is systematically biased by alterations in these brain areas. Specifically, apathetic patients’ hypersensitivity to effort may lead to them learning more rapidly associations between a course of action and its effort costs, leading apathetic patients to give up more quickly. Alternatively, a blunted sensitivity to rewarding outcomes may lead to a reduced willingness to learn that actions are associated with rewards and are worth pursuing. One recent study specifically examined outcome related activity in a gambling task in apathetic versus non-apathetic PD patients (Martinez-Horta et al., 2014). Apathetic PD patients showed a blunting of the typical feedback-related negativity, which putatively originates from the ACC and is thought to drive learning. This suggests that deficiencies in outcome processing in the ACC-VS circuit might be central to some aspects of apathy and highlights the potential for understanding the disorder through investigating motivation in these three different phases of behaviour.

In section two, we outlined evidence that the brain regions most associated with apathy across many neurological disorders – the VS and ACC – underlie motivated behaviour across three different phases. Notably, in a parallel line of work, motivational impairments including apathy have also been linked to similar underlying mechanisms and processes in psychiatric conditions (e.g. schizophrenia; (Holroyd and Umemoto, 2016). In this work, deficits in motivation and persistence during extended behaviours were linked to structural and functional changes in the ACC across different disorders. Taken together these frameworks suggest similar mechanisms may underlie apathy, regardless of the primary diagnosis, as a result of dysfunction in the VS and ACC.

A challenge for future research will be to determine the extent to which different aspects of apathy map onto the different aspects of behaviour that are supported by this system. Are motivational impairments tied to all aspects of this framework (i.e. motivating behaviour, persisting and learning what is worth doing) or is apathy characterized by a specific signature related to a deficit in this circuit (e.g. just the over-weighting of effort costs)? As noted in Section 2, the same regions of dACC and VS are implicated in each of the different phases of behaviour, but neurophysiological evidence points to partially distinct populations of neurons guiding them. Such hypotheses can be tested and falsified with precision by using the framework we have put forward here.

## 5 Conclusions

Here, we have outlined a framework for understanding apathy in terms of functional disruption of brain systems that play a key role in normal motivated behaviour. Crucially, this approach is not tied to the molecular pathology underlying the diverse diseases that lead to apathy. Instead, the premise here is that different pathologies can lead to a similar phenotype by disrupting common brain systems. Our review of neuroanatomical studies that have been conducted across disease types reveals that deficits in self-initiated, goal-directed motivated behaviour in apathy appear to be related to dysfunction of ACC, VS and connected structures, including OFC, VP and VTA. Furthermore, we have demonstrated that current knowledge of the normal roles of these regions in motivated behaviour – rooted within the framework of cost-benefit decision making – allows a clear, biologically plausible mechanistic approach to apathy, with testable predictions. We propose that this framework will provide new insights into the basis of apathy across pathology and the potential for novel treatments.
